# Supplementary material for: Bioinformatic Analysis of a Set of 14 Temperate Bacteriophages Isolated from Staphylococcus aureus Strains Highlights Their Massive Genetic Diversity
Source: Microbiol Spectr. 2022 Jul 26;10(4):e00334-22. doi: 10.1128/spectrum.00334-22 (PMC9430571; doi:10.1128/spectrum.00334-22)
Supplement: Supplemental file 1 — Supplemental material. Download spectrum.00334-22-s0001.pdf, PDF file, 1.7 MB [file spectrum.00334-22-s0001.pdf]

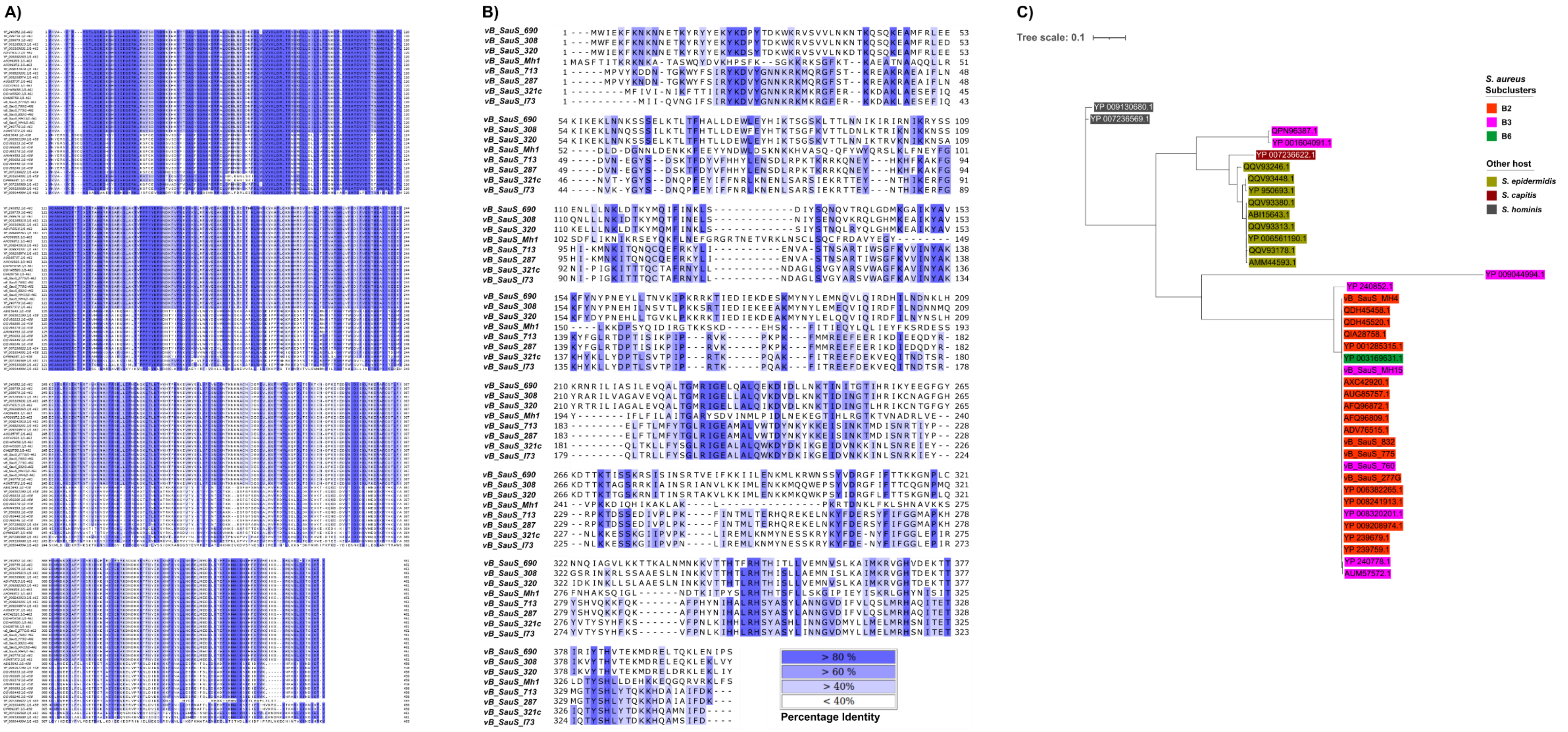

Figure S1 – Sequence comparison of the tyrosine and serine integrases. The protein sequences either from the serine A) and tyrosine integrases B) were aligned using MAFFT. The graphic representation was made with Jalview and were colored according to sequence percentage identity. C) The phylogenetic tree of the serin integrases were inferred by Maximum-Likelihood algorithm with the PhyML program and plotted in iTOL.

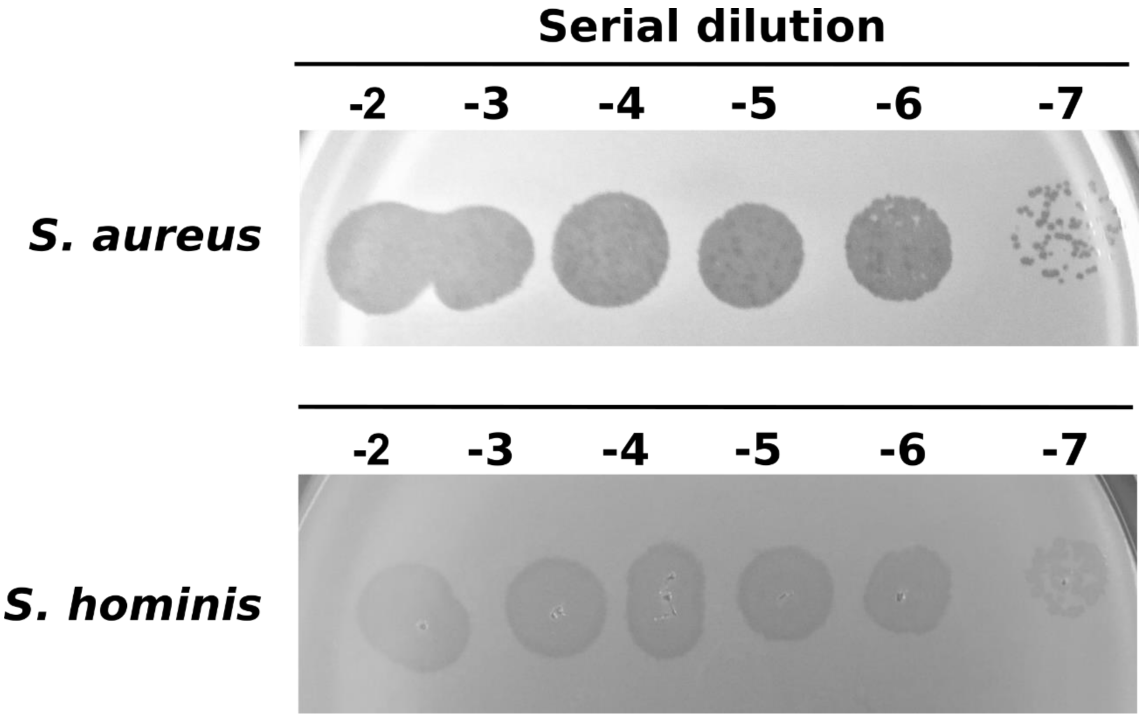

Figure S2. Propagation of vB_SauS_I73 on *Staphylococcus hominis* clinical strains. Six bacteriophages of our set, representing subclusters B2, B3, B5 and B7 were assayed for propagation (serial tenfold dilutions, -2 to -7) on CNS strains with negative results excepted for two *S. hominis* strains in which faint haloes and plaques were observed in all cases. Propagation of vB_Sau_S I73 on *S. aureus* RN4220 and *S. hominis* M15 strains is shown. Incubation times are 14 h and 48 h respectively
